# Upregulation of *miR-205* induces CHN1 expression, which is associated with the aggressive behaviour of cervical cancer cells and correlated with lymph node metastasis

**DOI:** 10.1186/s12885-020-07478-w

**Published:** 2020-10-27

**Authors:** Jianbing Liu, Yunfeng Li, Xihua Chen, Xiangbo Xu, Haoqi Zhao, Shufang Wang, Jianqing Hao, Bin He, Shuyan Liu, Jiedong Wang

**Affiliations:** 1 School of Basic Medical Sciences, Shanxi Medical University, Taiyuan, 030001 People’s Republic of China; 2 Basic Medical College, Hebei University of Chinese Medicine, Shijiazhuang, 050017 People’s Republic of China; 3 Reproductive Physiology Laboratory, National Research Institute for Family Planning, Beijing, 100081 People’s Republic of China; 4 School of Pre-clinical Sciences, Guangxi Medical University, Nanning, 530021 People’s Republic of China

**Keywords:** Cervical cancer, microRNA-205, a1-chimaerin, Migration, Invasion, Cancer gene

## Abstract

**Background:**

Cervical cancer is the leading cause of cancer-related death in women worldwide. However, the mechanisms mediating the development and progression of cervical cancer are unclear. In this study, we aimed to elucidate the roles of microRNAs and a1-chimaerin (CHN1) protein in cervical cancer progression.

**Methods:**

The expression of *miR-205* and CHN1 protein was investigated by in situ hybridisation and immunohistochemistry. We predicted the target genes of *miR-205* using software prediction and dual luciferase assays. The expression of mRNAs and proteins was tested by qRT-PCR and western blotting respectively. The ability of cell growth, migration and invasion was evaluated by CCK-8 and transwell. Cell apoptosis was analysed by flow cytometry analysis.

**Results:**

We found that *miR-205* and CHN1 were highly expressed in human cervical cancer tissue compared with paired normal cervical tissues. The *CHN1* gene was shown to be targeted by *miR-205* in HeLa cells. Interestingly, transfection with *miR-205* mimic upregulated CHN1 mRNA and protein, while *miR-205* inhibitor downregulated CHN1 in high-risk and human papilloma virus (HPV)-negative human cervical cancer cells in vitro,. These data suggested that *miR-205* positively regulated the expression of CHN1. Furthermore, the *miR-205* mimic promoted cell growth, apoptosis, migration, and invasion in high-risk and HPV-negative cervical cancer cells, while the *miR-205* inhibitor blocked these biological processes. Knockdown of CHN1 obviously reduced the aggressive cellular behaviours induced by upregulation of *miR-205*, suggesting that *miR-205* positively regulated CHN1 to mediate these cell behaviours during the development of cervical cancer. Furthermore, CHN1 was correlated with lymph node metastasis in clinical specimens.

**Conclusions:**

Our findings showed that *miR-205* positively regulated CHN1 to mediate cell growth, apoptosis, migration, and invasion during cervical cancer development, particularly for high-risk HPV-type cervical cancer. These findings suggested that dysregulation of *miR-205* and subsequent abnormalities in CHN1 expression promoted the oncogenic potential of human cervical cancer.

## Background

Cervical cancer is one of the most commonly diagnosed cancer and the leading causes of cancer-related death in women worldwide [1, 2]. However, despite the prevalence of this disease, therapeutic strategies are not sufficient, and patients with advanced disease are faced with poor outcomes.

MicroRNAs (miRNAs) are short (20–24 nt) noncoding RNAs that post-transcriptionally regulate gene expression in multicellular organisms by affecting both the stability and translation of mRNAs [3]. miRNAs regulate the expression of up to 60% of human genes [4] and generally reduce the protein expression of various targeted mRNAs [5]. Dysregulation of miRNA expression has been demonstrated in human cervical cancer tissues and cervical cancer cell lines; for example, *miR-10a*, *− 222*, *−196a*, *− 590*, *− 361-5p*, and *− 205* have been shown to promote cervical cancer cell growth, migration, and invasion [6–11], while *miR-214*, *−26a*, *− 218* and *− 205* have been shown to inhibit cancer cell growth, migration, and invasion [12–15]. Moreover, studies of human cervical cancer have shown that dysregulation of miRNAs regulates various cancer-related genes [8, 9, 16].

*MiR-205* has been shown to have dual functions as an oncogenic miRNA or tumour-suppressive miRNA, depending on cell context [5, 17]. Indeed, some studies have shown that *miR-205* serves as a tumour-suppressive miRNA by inhibiting the proliferation and invasion of cancer cells [12, 18–21], while other studies have shown that *miR-205* promotes tumour initiation, proliferation, and migration [11, 22]. Additionally, *miR-205* positively regulates transcriptional activation of the tumour-suppressor genes *interleukin (IL)-24* and *IL-32* in prostate cancer [21] and directly regulates *IL-24* in human KB oral cancer cells [23]. Interestingly, *miR-205* expression is upregulated in human cervical cancer tissues and cell lines [11, 24, 25], and serum *miR-205* levels are also increased in patients with cervical cancer [26]. Functionally, overexpression of *miR-205* has been shown to promote cell proliferation and migration by targeting the *CYR61* and *CTGF* genes [11]; however, these genes have not been shown to be associated with cancer. Therefore, the mechanisms through which *miR-205* mediates cervical cancer progression remain unknown.

n-Chimaerin (a1-chimaerin, CHN1) is a GTPase-activating protein that exhibits activity toward the small GTPase Rac [27]. CHN1 may play a role in mediating cell motility [28, 29]. Moreover, bioinformatics prediction has shown that CHN1 is a putative target of *miR-205* and a potential cancer-associated gene listed in the Cancer Gene Census [30]. Therefore, we hypothesised that CHN1 might be regulated by *miR-205* and involved in the development and metastasis of human cervical cancer.

In the current study, we aimed to determine the mechanisms through which *miR-205* mediates the progression and development of cervical cancer. To this end, we analysed the relationships between *miR-205* and CHN1 expression and function in human cervical cancer tissues and cell lines. Our data supported that CHN1 and *miR-205* might be biomarkers of human cervical cancer metastasis and potential therapeutic targets in human cervical cancer.

## Methods

### Tissue samples and human cervical carcinoma cell lines

Human cervical cancer tumours and adjacent non-tumour tissues were obtained from Guangxi Medical University (China). The clinicopathological characteristics of the samples are summarised in Table 1. A cervical cancer tissue microarray was purchased from Shanghai Outdo Biotech Co. Ltd. (China). All patients provided informed consent for the use of their tissues before surgery. The study was approved by the Ethics Committee of the National Research Institute for Family Planning.

**Table 1:** Statistical analysis of clinical samples

Clincal samples pathological characteristics	n	CHN1	CHN1 upregulated			***P***	Pearson Chi-Square
		–	+	++	+++		
**Tumor size (cm)**						0.660	1.597
≤ 3	18	2	9	4	3		
> 3	28	2	12	11	3		
**Differentiation grade**						0.269	7.594
I	2	1	1	0	0		
II	38	2	18	12	6		
III	6	1	2	3	0		
**Depth of invasion**						0.962	0.290
≤ 1/2 muscular layer	16	1	8	5	2		
>1/2 muscular layer	30	3	13	10	4		
**Tissue microarray lymphatic metastasis**						0.008	11.895
Absent	23	2	15	4	2		
Present	25	0	7	7	11		

- (0 ≤ HSCORE < 75); + (75 ≤ HSCORE < 150); ++(151 ≤ HSCORE < 225); +++(225 ≤ HSCORE ≤300)

The human cervical carcinoma cell lines HeLa, SiHa, and C33A were purchased from the Cell Resource Center of Peking Union Medical College (Beijing, China) and cultured in Dulbecco’s modified Eagle medium (DMEM) containing 10% foetal bovine serum (FBS), 100 IU/mL penicillin, and 10 mg/mL streptomycin. All cells were maintained at 37 °C in an atmosphere containing 5% CO_2_.

### In situ hybridisation of miR-205 with a digoxigenin (DIG)-labelled LNA probe

The sections (4 μm) of cervical cancer tissues and adjacent normal cervical tissues were treated with proteinase K (20 mg/mL) for 15 min and refixed in 4% PFA for 15 min. After acetylation with 0.25% acetic anhydride in 0.1 M triethanolamine (pH 8.0) for 10 min, sections were prehybridised with hybridisation buffer (Roche, Mannheim, Germany) at 40 °C for 2 h and then hybridised with a DIG-labelled LNA-*miR-205* probe (5′-CAG(+A)C(+T)CCGG(+T)GGAA(+T)GA(+A)GGA-DIG-3′) at 40°Covernight. The sections were then incubated in buffer containing anti-DIG-antibody (Roche) 2 h at 37 °C, followed by staining with NBT and BCIP (Promega, Madison, WI, USA). Samples were viewed under a Nikon TE 2000-U microscope (Nikon, Tokyo, Japan).

### Immunohistochemical analysis of CHN1

Sections (4 μm) of cervical cancer tissues and adjacent normal cervical tissues were dewaxed and rehydrated, followed by an antigen retrieval procedure (citrate buffer, pH 6.0; 95 °C heat for 15 min). For CHN1 staining, the sections were soaked in 3% H_2_O_2_ for 15 min and incubated overnight at 4 °C with rabbit anti-CHN1 antibodies (12048–1-AP; 1:150; Proteintech, USA). Matched rabbit nonimmune IgG was used as a negative control. The sections were then treated with horseradish peroxidase (HRP)-conjugated anti-rabbit IgG (PV-6001; Zymed Laboratories, China) and incubated for 20 min at 37 °C; the proteins were visualised with 3,3′-diaminobenzidine tetrahydrochloride and counter stained with hematoxylin. Immunohistochemical analysis of CHN1 was carried out according to the “HSCORE” method [31]; an HSCORE of 75 or greater was considered positive. The specimens were analysed by two observers who were unaware of the patients’ clinical outcome. Discrepancies between the observers were found in < 10% of the slides examined, and consensus was reached on further review.

### Transfection and cotransfection

The *miR-205* mimic, *miR-205* inhibitor, corresponding negative control (NC), and siRNA duplex against human CHN1 were designed and synthesised by GenePharma (GenePharma Co., Ltd., Shanghai, China). Their sequences are shown in Table 2. Transient transfection was performed using lipofectamine 2000 reagent (Invitrogen, Carlsbad, CA, USA) according to the manufacturer’s instructions.

**Table 2:** The sequences of siRNA, miRNA and primer used in this study

Gene	Sequence
CHN1 3’UTR	Forward: 5′-(C)TTGAGGGGAAAAGAAATG-3’
	Reverse: 5′-ATGTAACAGCCAGAGGTGC-3’
CHN1-mut	Forward: 5′-AGAAATGTTTTACAGGCTGGCCGATGTTTTATAG-3’
	Reverse: 5′-CGGCCAGCCTGTAAAACATTTCTTTTCCCCTCA-3’
Has-miR-205	RT-205: 5′-GTCGT ATCCA GTGCA GGGTC CGAGG TATTC GCACT GGATA CGACC AGACT-3’
	Forward: 5′-AATTGTCCTTCATTCCACCGG-3’
	Reverse: 5′-GTGCAGGGTCCGAGGT-3’
Human U6	Forward: 5′-CGCTTCGGCAGCACATATAC-3’
	Reverse: 5′-TTCACGAATTTGCGTGTCAT-3’
CHN1	Forward: 5′-GGAGCTACCTCATCCGGGAG-3’
	Reverse: 5′-TGTGTCTCTTTCAGGACTGGCA-3’
GAPDH	Forward: 5′-GGTCTTACTCCTTGG AGGCCATGTG-3’
	Reverse: 5′-ACCTAACTACATCGTTTACATGTT-3’
LNA-miR-205 probe	5′-CAG(+A)C(+T)CCGG(+T)GGAA(+T)GA(+A)GGA-Dig-3’
Hsa-miR-205	5′-UCCUUCAUUCCACCGGAGUCUG-3’
Hsa-miR-205 mimic	5′-GACUCCGGUGGAAUGAAGGAUU-3’
MircoRNA inhibitor NC	5′-CAGUACUUUUGUGUAGUACAA-3’
Hsa-miR-205 inhibitor	5′-CAGACUCCGGUGGAAUGAAGGA-3’
Negative control	5′-UUCUCCGAACGUGUCACGUTT-3’
si-CHN1	5′-GGCUUGAUUACUCUCUAUATT-3’

### Plasmid constructs and dual-luciferase activity assay

The 3′ untranslated regions (UTRs) of the human *CHN1* gene (NM_001025201.2) were amplified by polymerase chain reaction (PCR) from human genomic DNA, cloned into the *Sbf*I and *Nhe*I site of the pmirGLO Dual-Luciferase miRNA Target Expression Vector (Promega), checked for orientation, and sequenced; the resulting plasmid was named pmirGLO-CHN1-wt. PCR primers used to amplify the *CHN1* 3’UTR are shown in Table 2. Site-directed mutagenesis of the *miR-205* target site in the *CHN1* 3’UTR was carried out using an Easy Mutagenesis System (Transgen, China), with pmirGLO-CHN1-wt as a template; the resulting plasmid was named pmirGLO-CHN1-mut.

Next, 5 × 10^4^ cells were seeded in each well of a 48-well plate at 24 h before transfection. For reporter assays, the cells were transiently cotransfected with 0.25 μg wild-type (WT) or mutant reporter plasmid and 7.5 pmol NC or *miR-205* mimic using Lipofectamine 2000. At 48 h after cotransfection, Firefly and *Renilla* luciferase activities were measured consecutively using Dual Luciferase Assays (Promega) according to the manufacturer’s instructions. Three independent experiments were performed.

### qRT-PCR

Total RNA extraction and qRT-PCR experiments were performed for analysis of gene expression as follows. Briefly, RNA was isolated using TRIzol reagent (Invitrogen). For cDNA synthesis, approximately 2 μg of total RNA was used for reverse transcription with oligo-(dT)_18_ primers using moloney murine leukaemia virus (M-MLV) reverse transcriptase (TaKaRa Bio, Otsu, Japan). The specific forward primer for *miR-205* was designed by GenePharma based on the miRNA sequence from the miRbase database.

qRT-PCR was performed with an ABI Prism 7700 Sequence Detector System (PE Applied Biosystems, Foster City, CA, USA) using SYBR Premix Ex Taq II (TaKaRa Bio) and specific primers for each gene. To control for uniform amount of input RNA template, mRNA and miRNA expression results were normalised to the expression level of the internal control gene *GAPDH* or *U6* snRNA, respectively. Thermal cycling conditions were as follows: an initial activation cycle at 95 °C for 30 s, followed by 40 cycles of denaturation (95 °C for 10 s), annealing, and amplification (60 °C for 30s). The final amplification products were verified by agarose gel electrophoresis for treatment samples and negative controls. The primer sequences are shown in Table 2. Each sample was assayed in triplicate. To compare the expression levels among different samples, relative quantification was achieved using the 2^-ΔΔCt^ approach, in which ΔΔCt is the calibrated Ct value.

### Western blot analysis

Total protein lysates were obtained using RIPA lysis buffer supplemented with 1 mM PMSF, protease inhibitor cocktail, 1 mM Na_3_VO_4_, and 10 mM NaF (Sigma Aldrich, St. Louis, MO, USA). The protein concentrations in extracts were determined by colorimetric BCA protein assays (Thermo Scientific, USA). Proteins were separated by SDS-polyacrylamide gel electrophoresis (PAGE) on 10% Tris-glycine gels (Amresco, Solon, OH, USA) and then transferred onto polyvinylidene fluoride membranes (Millipore, Billerica, MA, USA). Membranes were blocked for 1 h at RT with TBST (50 mM Tris-HCl, 150 mM NaCl, and 0.1% [v/v] Tween-20) containing 5% (w/v) nonfat dried milk and were subjected to immunoblotting with antibodies to CHN1 (12048–1-AP; Proteintech) and β-actin (CoWin, Beijing, China).

### Cell proliferation assay

The proliferation of HeLa, SiHa, and C33A cells was estimated with a Cell Counting Kit-8 (CCK-8; Dojindo Laboratories, Japan) according to the manufacturer’s instructions following transfection with *miR-205* mimic, NC, *miR-205* inhibitor, or inhibitor NC, with five wells for each treatment. The experiment was repeated three times, and the results are described as the ratio of the absorbance at 450 nm for the *miR-205* mimic or inhibitor to that of the corresponding control.

### Flow cytometry analysis

Cell apoptosis was analysed using flow cytometry analysis with an Alexa Fluor 488 annexin V/Dead Cell Apoptosis Kit (Invitrogen). Samples containing 5 μL Alexa Fluor 488 annexin V and 1 μL of 100 μg/mL propidium iodide (PI) were assayed to determine the phosphatidylserine (PS) exposure on the outer leaflet of the plasma membrane. After incubation for 15 min at in a light-protected area, the specimens were quantified by flow cytometry (BD Biosciences, San Jose CA, USA). Each treatment was repeated twice, and the experiment was repeated three times.

### In vitro migration and invasion assays

HeLa, SiHa, and C33A cells were transfected with the *miR-205* mimic, NC, *miR-205* inhibitor, or inhibitor NC. Transfected cells were harvested and subjected to the following assays at 48 h after transfection. For migration assays, the transfected cells (0.5 × 10^6^ cells/mL) were seeded in the top of a chamber containing a membrane with 8.0-μm pores (Corning Costar Corp., Cambridge, MA, USA). Following a 12–18 h incubation period, cells that passed through the membrane were fixed and stained with hematoxylin. Cells were scraped and removed from the top of chamber. Membranes were mounted on cover slides, and cells were counted. Cell migration was quantified by counting the number of cells passing through the pores from five different randomly selected fields of view per sample at 100× magnification under a microscope. For invasion assays, Matrigel (BD Biosciences) diluted to 1 mg/mL in serum-free cold cell culture medium was added to the top of a chamber containing a membrane with 8.0-μm pores and incubated at 37 °C overnight until the matrigel solidified. Analysis was then carried out as described above. Samples were viewed under a Nikon TE 2000-U microscope (Nikon, Tokyo, Japan).

### Statistical analysis

Data are expressed as means ± SEMs. The statistical significance of the quantitative data was assessed by paired Student’s t-tests, and clinical correlations were analysed by Pearson chi-square test. *P* < 0.05 was considered to be statistically significant.

## Results

### *miR-205* and CHN1 expression levels were upregulated in human cervical cancer tissues

**Fig. 1:**
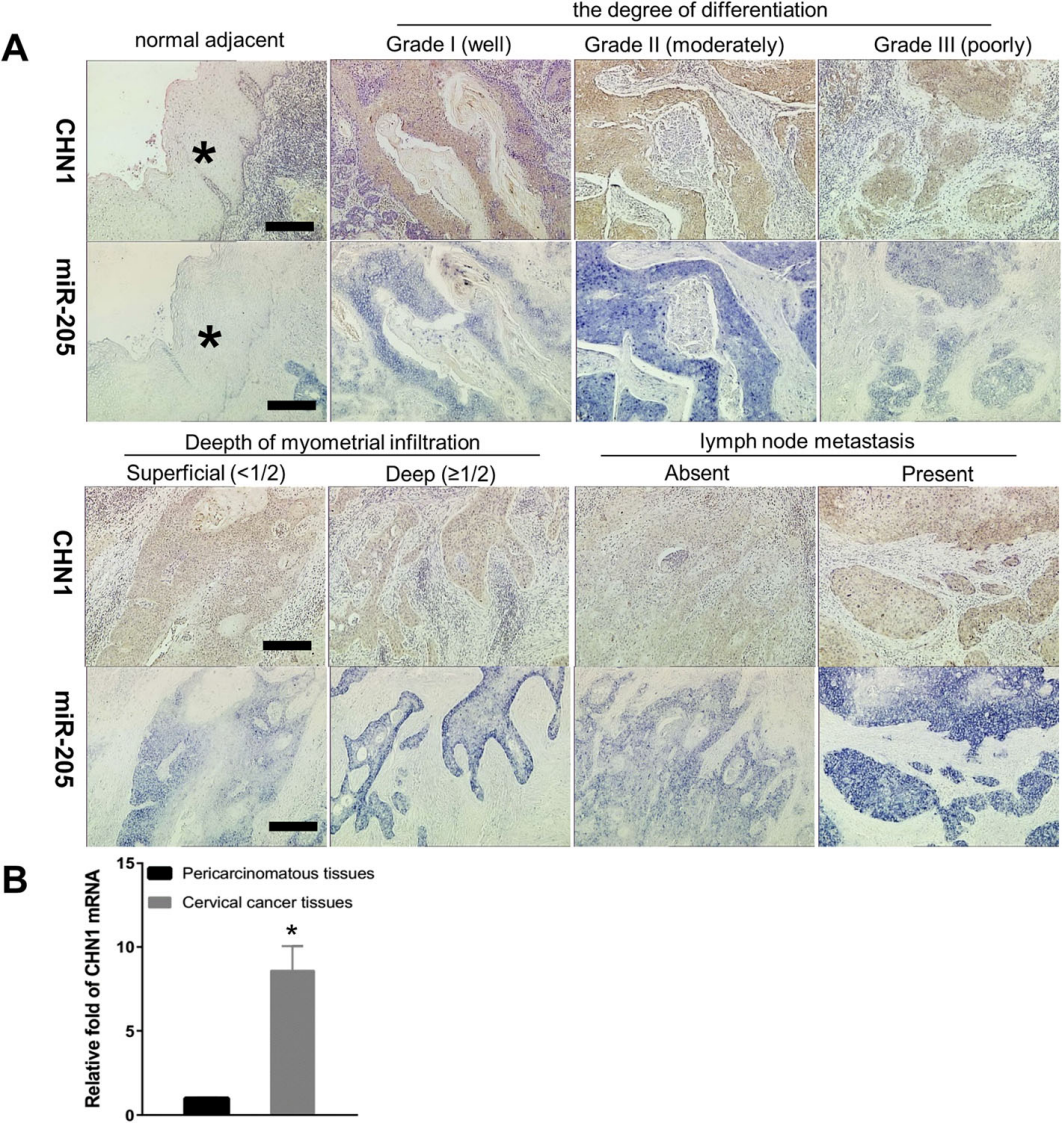
The expression of *miR-205* and CHN1 in cervical cancer. **a**:Distribution and expression of *miR-205* and CHN1 protein in human cervical tissues. The expression and localisation of *miR-205* in human cervical cancer tissues and adjacent normal cervical tissues was determined using in situ hybridisation. The stain was developed with BCIP/NBT. The expression and localisation of CHN1 protein in human cervical cancer tissues and adjacent normal cervical tissues was analysed by immunohistochemistry. The stain was developed with DAB, and nuclei were stained with hematoxylin. *Normal cervical epithelial tissue adjacent to carcinoma. Bar = 200 μm. **b**: The expression of *CHN1* mRNA in the cervical cancer tissues and the para-carcinoma tissues was detected by qRT-PCR. *GAPDH* served as an internal reference gene. *P < 0.05

**Fig. 2:**
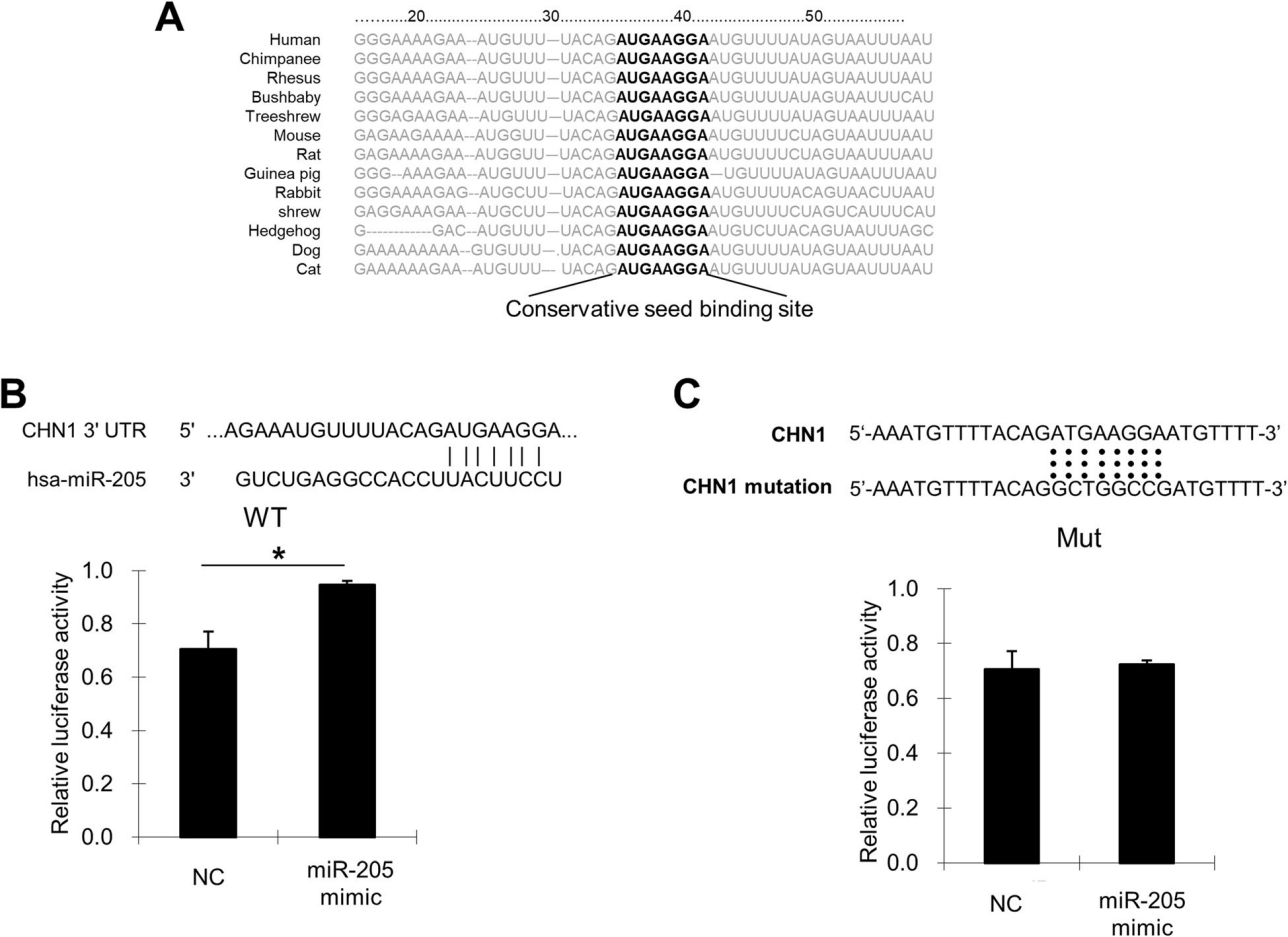
Prediction and detection of *miR-205* targets. **a** *miR-205* binding sites in the 3′UTR of *CHN1* in cross-species. **b** Confirmation of the *miR-205* target gene. HeLa cells were cotransfected with negative control (NC), *miR-205* mimic, and pmirGLO-CHN1-wt for dual luciferase assays. PRL-TK, containing *Renilla* luciferase, was cotransfected with the 3′UTR of *CHN1* for data normalisation. **c** Mutation of the *miR-205* binding site. HeLa cells were cotransfected with NC, *miR-205* mimic, and pmirGLO-CHN1-mt for dual luciferase assays. *P < 0.05

Expression patterns of *miR-205* were analysed by in situ hybridisation in 46 pairs of human cervical cancer tissues and adjacent normal cervical tissues. As shown in Fig. 1, the expression of *miR-205* was high in human cervical cancer tissues, but low or undetectable in normal cervical epithelium and noncancerous cervical stratified epithelium. Then, *miR-205* target genes were searched using bioinformatic prediction of potential *miR-205* binding sites with PicTar, Targetscan, miRanda, and miRBase programs, to elucidate the molecular mechanisms through which *miR-205* functioned in human cervical carcinogenesis. Among the identified targets, CHN1 attracted our attention for following reasons: (i) there were two binding sites for *miR-205* in the 3’UTR of *CHN1*, one of which was highly conserved among different species (Fig. 2a); and (ii) *CHN1* has been reported to be a cancer-associated gene [26] involved in cell migration and cancer cell motility [24, 28]. CHN1 mRNA expression was elevated in the cervical cancer tissues than in the adjacent tissues of cancer (Fig. 1b). CHN1 protein expression was abnormally increased in tumour cell masses/cords, but was barely detectable or undetectable in normal cervical epithelium and noncancerous cervical stratified epithelium (Fig. 1a). Notably, in locations where *miR-205* expression was enhanced, CHN1 was also increased; conversely, when *miR-205* expression was decreased, CHN1 was also decreased. These data suggested that *miR-205* and CHN1 were synchronously upregulated in human cervical tumours, which was contradictory to our expectations.

### Validation of *CHN1* as target of *miR-205* in human cervical cancer cells

According to software-based prediction, the first seven nucleotides at the 5′-end of *miR-205* were complementary to nucleotides 36–43 of the 3’UTR of the *CHN1* gene (seed sequence; Fig. 2b). To validate whether *CHN1* was a target of *miR-205*, HeLa cells were cotransfected with pmirGLO-CHN1-wt and *miR-205* mimic or NC. Compared to the NC, transfection with the *miR-205* mimic increased the relative luciferase activity of the pmirGLO-CHN1-wt construct (*P* < 0.05; Fig. 2b). Moreover, cotransfection of HeLa cells with a construct harbouring a mutated seed sequence and the NC or *miR-205* mimic revealed that the *miR-205* mimic could not increase the relative luciferase activity of the pmirGLO-CHN1-mut construct in the absence of the WT seed sequence (Fig. 2c). These results strongly supported that *CHN1* was a target of *miR-205* in HeLa cells.

### *miR-205* positively regulated the expression of CHN1 in human cervical cancer cells in vitro

**Fig. 3:**
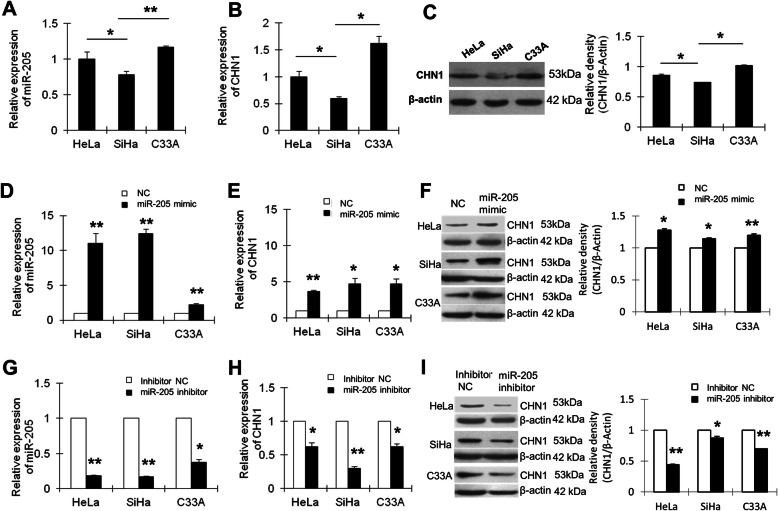
Confirmation of the relationship between *miR-205* and CHN1. **a** The expression of *miR-205* in HeLa, SiHa, and C33A cells was detected by qRT-PCR. U6 served as an internal reference and was used to normalise *miR-205* expression. The y-axis displays the relative expression of *miR-205* normalised to the expression of U6. **b** The expression of *CHN1* mRNA in HeLa, SiHa, and C33A cells was detected by qRT-PCR. *GAPDH* served as an internal reference gene. **c** CHN1 protein expression was detected by western blotting. β-Actin was used as a loading control. The black histogram shows the optical densities of the signals quantified by densitometric analysis and represented as the CHN1 intensity/β-Actin intensity for normalisation of gel loading and transfer. **d** HeLa, SiHa, and C33A cells were transfected with NC or *miR-205* mimic. The expression of *miR-205* was detected by qRT-PCR. **e** The level of *CHN1* mRNA was detected by qRT-PCR. *GAPDH* served as an internal reference gene. **f** CHN1 protein expression was detected by western blotting. β-Actin was used as a loading control. **g** HeLa, SiHa, and C33A cells were transfected with inhibitor NC or *miR-205* inhibitor. The expression of *miR-205* was detected by qRT-PCR. **h** *CHN1* mRNA expression was detected by qRT-PCR after transfection of cells with inhibitor NC or *miR-205* inhibitor. *GAPDH* served as an internal reference gene. **i** CHN1 protein expression was detected by western blotting after transfection of cells with inhibitor NC or *miR-205* inhibitor. β-Actin was used as a loading control. *P < 0.05, **P < 0.01. The cropping of the blot was done. Full-length uncropped blots are presented in Supplementary Figure 1, which all the samples derived from the same experiment and blots were processed in parallel

As shown in Fig. 3a and b, the expression levels of *miR-205* and CHN1 mRNA were higher in C33A cells and lower in SiHa cells than those in HeLa cells. The results of western blotting analysis of CHN1 protein were consistent with these results (Fig. 3c, Supplementary Figure 1). Thus, overall, these results demonstrated that high *miR-205* expression was associated with high CHN1 expression, and vice versa, in the cervical cancer cell lines. The levels of *miR-205*, *CHN1* mRNA, and CHN1 protein were significantly increased in cells transfected with *miR-205* mimic compared with those in cells transfected with NC (Fig. 3d–f, Supplementary Figure 1). Conversely, the levels of *miR-205*, *CHN1* mRNA, and CHN1 protein were reduced in cells transfected with *miR-205* inhibitor, in comparison to those in cells transfected with inhibitor NC (Fig. 3g–i, Supplementary Figure 1). These results demonstrated that *miR-205* positively and directly regulated the expression of CHN1 in human cervical cancer cells.

### *miR-205-*dependent expression of CHN1 was involved in the proliferation, apoptosis, migration, and invasion of human cervical cancer cells in vitro

**Fig. 4:**
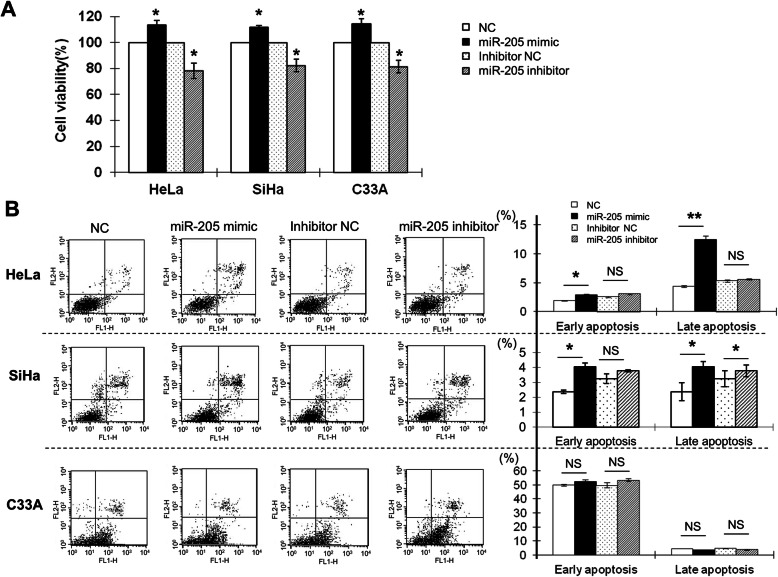
The effects of *miR-205* on the proliferation and apoptosis of human cervical cancer cells. **a** HeLa, SiHa, and C33A cells were transfected with the NC, *miR-205* mimic, inhibitor NC, or *miR-205* inhibitor. At 48 h after transfection, cell proliferation was determined by CCK-8 assay. All experiments were performed at least three times, and cell proliferation was determined as the stimulation index (SI; i.e., the ratio of absorbance at 450 nm of cells transfected with *miR-205* mimic or inhibitor to that of cells transfected with NC or inhibitor NC). **b** HeLa, SiHa, and C33A cells were transfected with the NC, *miR-205* mimic, inhibitor NC, or *miR-205* inhibitor for 48 h. Cells were then stained with annexin V/PI and subjected to flow cytometry analysis. Lower left quadrant, viable cells (annexin V-FITC and PI negative); lower right quadrant, early apoptotic cells (annexin V-FITC positive and PI negative); upper right quadrant, late apoptotic/necrotic cells (annexin V-FITC and PI positive). The average percentage of apoptotic cells was analysed in cells transfected with *miR-205* mimic or inhibitor at early and late stages. The histograms represent the average percentages of apoptotic cells in cells transfected with *miR-205* mimic at early and late stages or *miR-205* inhibitor at early and late stages. The experiment was repeated at least three times. *P < 0.05, **P < 0.01, NS: not significant

The functions of *miR-205*-dependent CHN1 expression in the pathological processes of cervical cancer cells were examined. At 48 h after transfection, compared to those in cells transfected with NC (Fig. 4a), the relative proliferation rates of HeLa, SiHa, and C33A cells transfected with *miR-205* mimic were increased by about 13.38% (*P* < 0.05), 11.77% (*P* < 0.05), and 14.28% (*P* < 0.05), respectively. Conversely, the relative proliferation rates in HeLa, SiHa, and C33A cells transfected with *miR-205* inhibitor were decreased by about 21.72% (*P* < 0.05), 17.56% (*P* < 0.05), and 18.62% (*P* < 0.05), respectively, in comparison with those in cells transfected with inhibitor NC (Fig. 4a). These results showed that overexpression of *miR-205* significantly promoted cervical cancer cell proliferation, while downregulation of *miR-205* suppressed cervical cancer cell proliferation.

Analysis of apoptosis revealed that transfection with the *miR-205* mimic significantly increased early apoptosis in HeLa (*P* < 0.05) and SiHa cells (*P* < 0.05), but not in C33A cells (*P* > 0.05). Late apoptosis was also enhanced in HeLa (*P* < 0.01) and SiHa cells (*P* < 0.05), but not in C33A cells (*P* > 0.05), as compared with that in cells transfected with NC (Fig. 4b). Interestingly, transfection with *miR-205* inhibitor did not affect early apoptosis in any of the three cell lines, while late apoptosis was increased in SiHa cells (*P* < 0.05), but not in HeLa or C33A cells (*P* > 0.05), as compared with those in cells transfected with inhibitor NC (Fig. 4b).

**Fig. 5:**
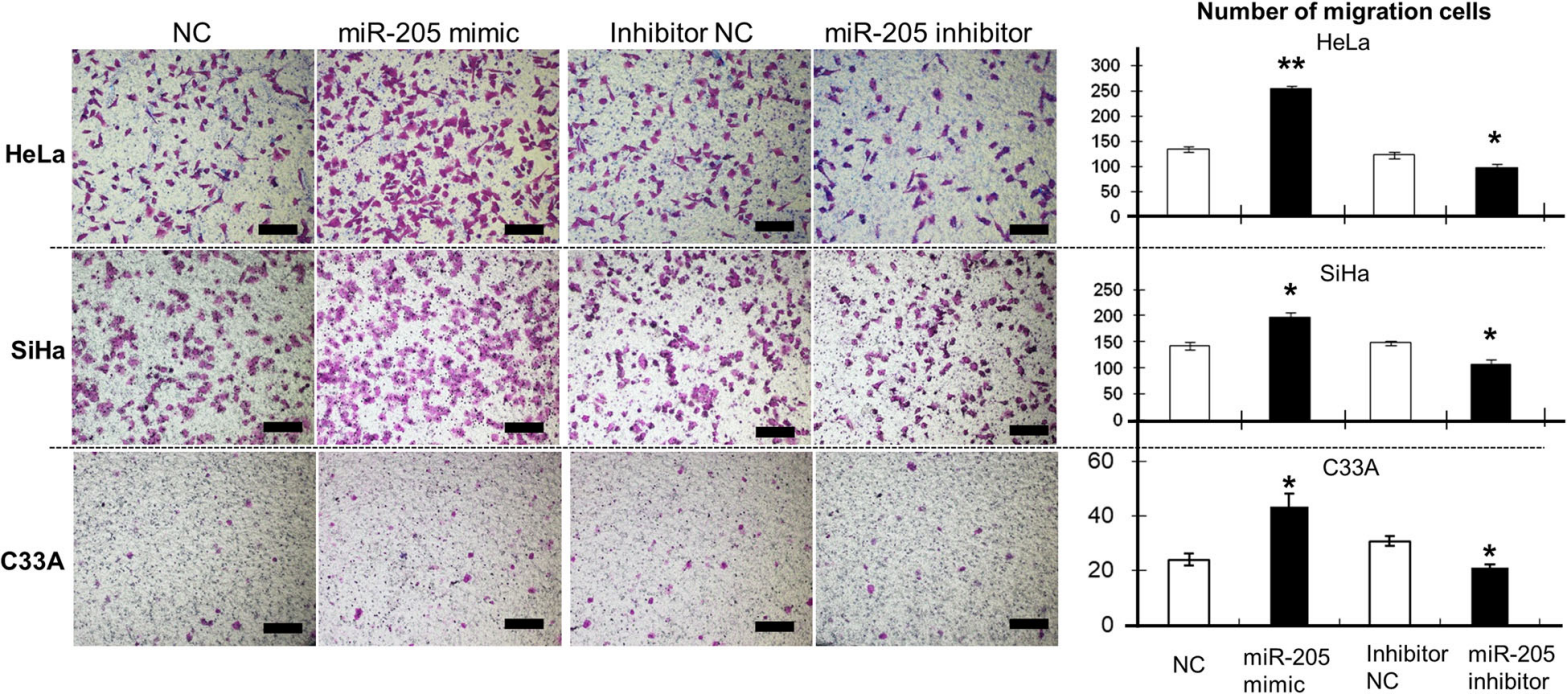
The effects of *miR-205* on the migration of human cervical cancer cells. HeLa, SiHa, and C33A cells were transfected with the NC, *miR-205* mimic, inhibitor NC, or *miR-205* inhibitor for 48 h. Cells were then subjected to migration assays as described in the Methods. Cell migration was quantified by counting the number of cells passing through the membrane from five different randomly selected fields of view per sample at 100× magnification. Representative images are shown. The histograms represent the number of migrated HeLa, SiHa, and C33A cells. Data are expressed as the means of independent triplicate experiments. Magnification, 100×; scale bar =200 μm. *P < 0.05, **P < 0.01

**Fig. 6:**
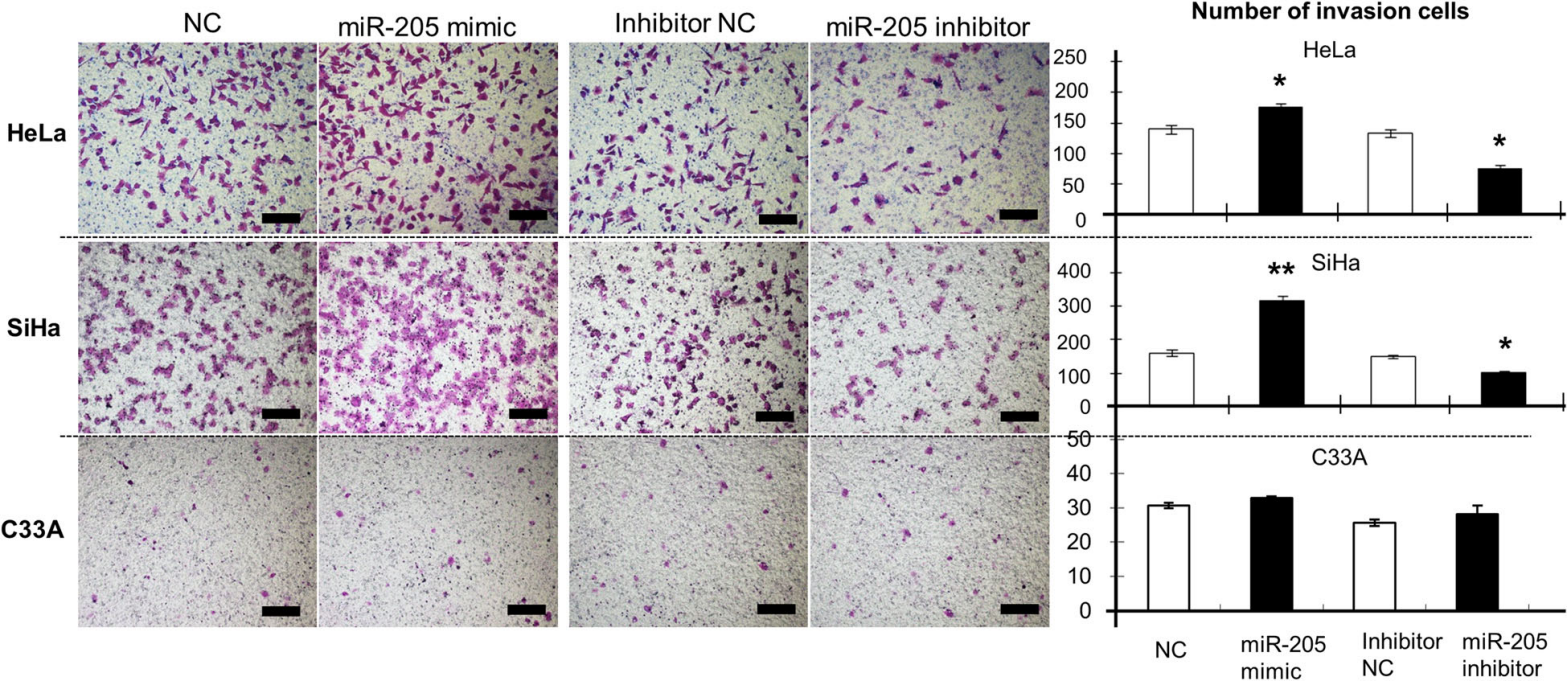
The effects of *miR-205* on the invasion of human cervical cancer cells. Cell invasion was quantified by counting the number of cells passing through the membrane from five different randomly selected fields of view per sample at 100× magnification. Representative images are shown. The histograms represent the number of invaded HeLa, SiHa, and C33A cells. Data are expressed as the means of independent triplicate experiments. Scale bar =200 μm. *P < 0.05, **P < 0.01

The migration capacity of HeLa, SiHa, and C33A cells transfected with the *miR-205* mimic was significantly higher than that in cells transfected with NC (HeLa, *P* < 0.01; SiHa and C33A, *P* < 0.05; Fig. 5). Similarly, the migration capacity was decreased in HeLa, SiHa, and C33A cells transfected with *miR-205* inhibitor, as compared with that in cells transfected with inhibitor NC (*P* < 0.05 for all cell lines; Fig. 5). Additionally, the invasive ability of HeLa and SiHa cells (HeLa, *P* < 0.05; SiHa, *P* < 0.01), transfected with the *miR-205* mimic was evidently enhanced, but C33A cells (*P* > 0.05), as compared with that in cells transfected with the NC (Fig. 6). Conversely, transfection with the *miR-205* inhibitor reduced invasive ability in HeLa and SiHa cells (HeLa and SiHa, *P* < 0.05), but not in C33A cells (*P* > 0.05), compared with that in cells transfect with the inhibitor NC (Fig. 6). These results suggested that *miR-205* expression might be closely associated with the metastasis of cervical cancer cells.

**Fig. 7:**
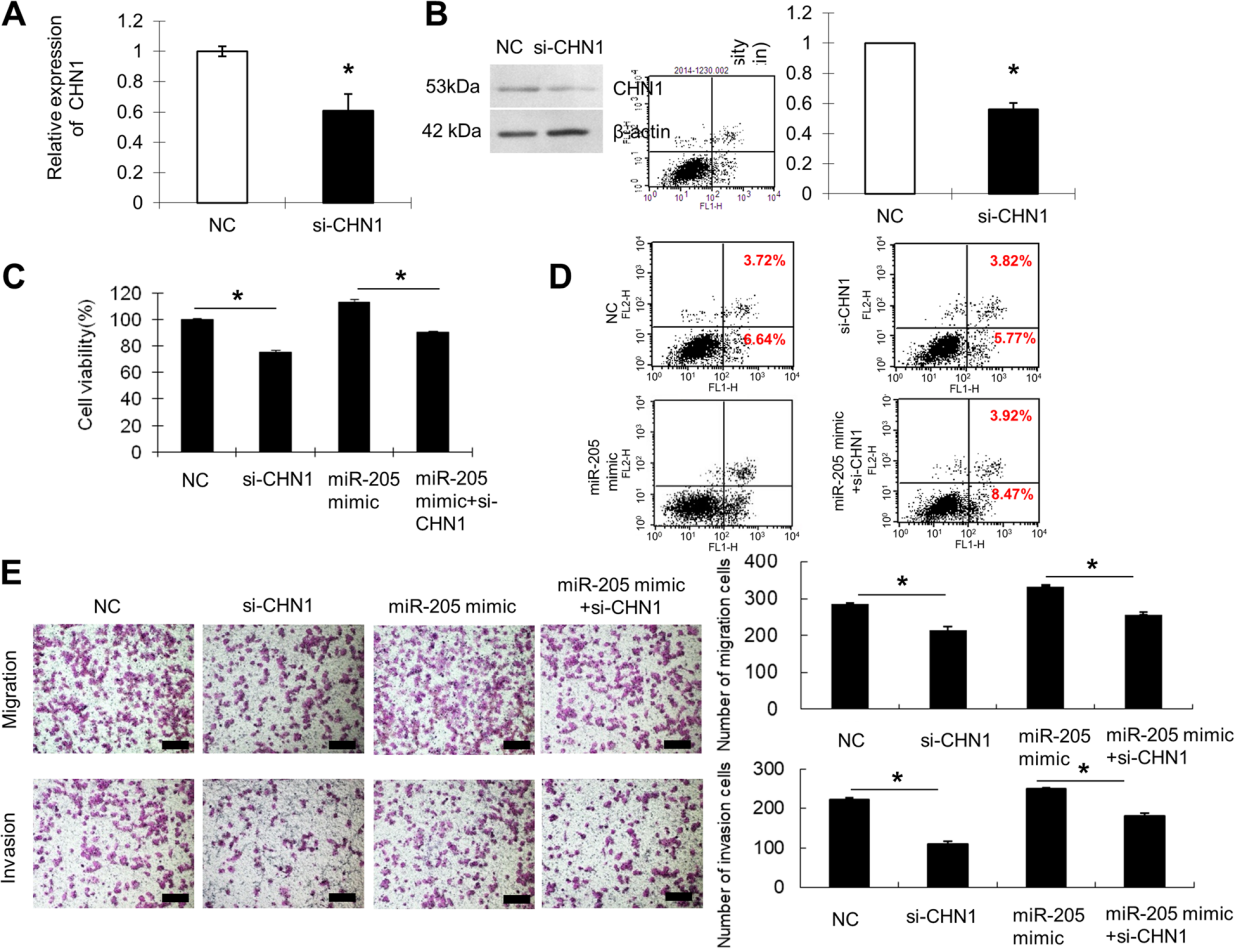
Knockdown of CHN1 attenuated the *miR-205*-mediated enhancement of cell growth and metastasis. **a** SiHa cells were transfected with NC or si-CHN1. At 48 h after transfection, *CHN1* mRNA expression was determined by qRT-PCR. GAPDH served as an internal reference gene. **b** CHN1 protein levels were determined by western blotting at 48 h after transfection. β-Actin was used as a loading control. The cropping of the blot was done. Full-length uncropped blots are presented in Supplementary Figure 2, which all the samples derived from the same experiment and blots were processed in parallel. **c** Cell proliferation was determined by CCK-8 assay at 48 h after transfection. SiHa cells were transfected with NC, si-CHN1, or *miR-205* mimic or cotransfected with *miR-205* mimic and si-CHN1. **d** Apoptosis was detected by flow cytometry. The percentages of early and late apoptotic cells (representative of three separate experiments) are shown in the lower right and upper right panels, respectively. **e** Cells (0.5 × 10^6^ cells/mL) were subjected to migration or invasion assays as described in the methods. Magnification, 100×; scale bar =200 μm. *P < 0.05

To further examine whether *miR-205* may have oncogenic roles through targeting of CHN1, the effects of CHN1 knockdown on *miR-205*-mediated cell behaviours were investigated. Importantly, transfection with si-CHN1 significantly downregulated CHN1 mRNA and protein expression (*P* < 0.05; Fig. 7a and b, Supplementary Figure 2). Functional analysis revealed that knockdown of CHN1 reduced cell viability, migration, and invasion (*P* < 0.05 for all) in SiHa cells (Fig. 7c and e). In addition, knockdown of CHN1 using si-CHN1 decreased the rate of apoptosis in SiHa cells (Fig. 7d). Finally, cotransfection of SiHa cells with si-CHN1 and the *miR-205* mimic, resulted in lower capacity for proliferation, migration, invasion, and apoptosis (*P* < 0.05 for all) than those in cells transfected with the *miR-205* mimic (Fig. 7c–e). These results demonstrated that the effects of *miR-205* overexpression on cell growth and metastasis were partially attenuated by knockdown of CHN1.

### CHN1 overexpression was associated with metastasis in human cervical cancer

The immunohistochemical analysis of CHN1 of 46 human cervical cancer samples were performed, to investigate the relationship between CHN1 overexpression and the clinical characteristics of human cervical cancer. The results showed that upregulation of CHN1 was not significantly associated with tumour size (*P* = 0.660), differentiation grade (*P* = 0.269), or depth of invasion (*P* = 0.962), suggesting that upregulation of CHN1 was a common characteristic in human cervical cancer (Table 1.). Therefore, CHN1 may be a novel biomarker for the clinical diagnosis of carcinogenesis of normal cervical epithelium.

To further analyse the differences between CHN1 expression and metastasis of cervical tumours, we investigated the expression of CHN1 in a microarray of cervical cancer tissues with or without lymph node metastasis. We found that the intensity of CHN1 expression was significantly associated with lymph node metastasis of cervical cancer (*P* = 0.008).

## Discussion

In this study, for the first time, we demonstrated that *miR-205* positively regulated the expression of CHN1 in cervical cancer and that *miR-205*-dependent upregulation of CHN1 promoted the proliferation, migration, and invasion of cervical cancer.

In previous studies, *miR-205* has been shown to have dual roles in cancer development, acting as either a tumour suppressor or oncogene. For example, *miR-205* expression was reduced in melanoma [32], oesophageal cancer [33], and oesophageal squamous cell carcinoma [34], but increased in endometrial adenocarcinoma [35], head and neck squamous cell carcinoma cell lines [36], and ovarian cancer [37]. In cervical cancer patients, serum *miR-205* was significantly upregulated, compared with that in healthy donors, and a high level of *miR-205* expression was correlated with poor tumour differentiation, lymph node metastasis, and increased tumour stage [26]. However, miR-205 was downregulated in cervical intraepithelial neoplasia and cervical squamous cell carcinoma [38]. In this study, we found that *miR-205* was highly expressed in cervical cancer samples compared to normal cervical tissues, demonstrating that *miR-205* functioned as an oncogene in this context.

*MiR-205* functions by regulating the expression of a variety of target genes. Contradictory to the traditional negative regulation observed in miRNAs, we found that *miR-205* positively regulated CHN1. Unlike the general mechanism of miRNA, some previous studies have shown that miRNAs can positively regulate target genes [21, 23, 39]. Specifically, early in 2007, the study published in *Science* showed that human *miR-369-3* directed association of Argonaute (AGO) and fragile X mental retardation-related protein 1 (FXR1) with the AU-rich elements (AREs) to switch from repression to activation on the translation of target gene mRNAs [39]. Further, *miR-205* in the KB oral cancer cells induced tumour suppressor gene IL-24 mRNA and protein by targeting its promoter [23]. Moreover, in prostate cancer, *miR-205* also upregulated the tumour suppressor genes *IL-24* and *IL-32* mRNA and protein by targeting their promoters [21]. In our study, *miR-205* induced the expression of CHN1 mRNA and protein by binding to the 3’UTR of CHN1. However, the target region observed in this study were different from that reported in previous studies. Further studies are needed to fully elucidate the mechanisms through which some miRNAs positively regulate target genes.

Cancer cell proliferation, migration, and invasion are characteristics of cancer development and essential for invasion of cancer cells into the lymph and blood vessels for development of metastatic lesions. Here, we found that *miR-205* was expressed in HeLa (HPV18^+^), SiHa (HPV16^+^), and C33A (HPV^−^) cells, contrary to a previous study showing that *miR-205* was not expressed in these three cell lines [25]. We also found that *miR-205* was vital for cell proliferation, invasion, and migration in the different of HPV types. These results were consistent with a previous study which showed *miR-205* could promote cell proliferation and migration [11]. *MiR-205* was also involved in cellular invasion in other types of cancer [34, 40]. Here, for the first time, we found that *miR-205* exhibited a novel function in cell invasion of cervical cancer. Additionally, our studies showed that knockdown of CHN1 in SiHa cells blocked cancer-associated biological processes, further highlighting the importance of *miR-205* and *miR-205-*dependent expression of CHN1 in cervical cancer development.

The expression levels and functions of *miR-205*/CHN1 varied in cervical cancer cell lines exhibiting different HPV types which are vital to cancer development. For example, *miR-205* was highly expressed in HPV-negative C33A cells, but did not function in cell apoptosis and invasion, as was observed for HeLa and SiHa cells (high-risk HPV18^+^ and HPV16^+^ cell lines, respectively). These data improved our understanding of the importance of HPV, particularly high-risk HPV infection, in the development of cervical cancer.

The interaction between HPV and miRNAs during carcinogenesis may be used to explain the difference of *miR-205*/CHN1 expression and function in the different cell lines. For instance, some miRNAs localise the sites of HPV insertion, while proteins encoded by HPV can act on miRNAs. Additionally, E family viral proteins of HPV modulate the expression of DNA methyltransferases to regulate gene expression [41]. Therefore, further studies are required to determine how different HPV types affect the expression and function of *miR-205*/CHN1 in cervical cancer.

In this study, we found that both CHN1 and *miR-205* functioned as oncogenes in cervical cancer. Therefore, it seems reasonable that *miR-205* positively regulated CHN1. Moreover, a previous study in HeLa indicated that *miR-205* mimic could increase the expression of F-actin, which is involved in cell migration and cell adhesion to the extracellular matrix [42]; however, F-actin is not a direct target of *miR-205*. Interestingly, microinjected CHN1 colocalised in situ with F-actin in Swiss3T3 and neuroblastoma cells [28]. Therefore, regarding our current results, *miR-205* may directly regulate CHN1 to affect F-actin expression, indirectly supporting the positive regulation of CHN1 by *miR-205* in cervical cancer.

Interestingly, overexpression of CHN1 was not significantly associated with tumour size, the degree of tumour differentiation, or the depth of invasion in cervical cancer specimens, potentially due to the small number of clinical samples investigated. Furthermore, in differentiated squamous cell carcinoma II, the expression of CHN1was increased in cancers with lymph node metastasis compared with that in cancers without lymph node metastasis, indicating that CHN1 is associated with lymph node metastasis and may be a potential molecular marker of tumour metastasis. As this is the first study to demonstrate the role of CHN1 in cervical cancer, further studies are required to validate these results.

In summary, *miR-205* was specifically upregulated in cervical cancer, and positively regulated CHN1 to control cancer cell proliferation, invasion, and migration. These findings improved our understanding of the mechanisms of positive regulation by miRNAs and suggested that *miR-205* and CHN1 might play a role as a novel predictive biomarker for clinical outcomes in cervical cancer.

## Conclusions

Our results have demonstrated that CHN1 and *miR-205* might be used as biomarkers of human cervical cancer metastasis and potential therapeutic targets, and *miR-205* positively regulated the expression of CHN1 in human cervical cancer.

## Supplementary information

**Additional file 1 : Supplementary Figure 1.** The original drawing of the cropped blots in the Fig. 3. Red dashed line showed where the cut was made.

**Additional file 2 : Supplementary Figure 2.** The original drawing of the cropped blots in the Fig. 7. Red dashed line showed where the cut was made.

## Data Availability

All data generated or analysed during this study are included in this published article and its supplementary information files.

## Abbreviations

CHN1: a1-chimaerin
HPV: Human papilloma virus
miRNAs: microRNAs
IL: Interleukin
DMEM: Dulbecco’s modified eagle medium
foetal FBS: Bovine serum
DIG: Digoxigenin
UTRs: Untranslated regions
PCR: Polymerase chain reaction
WT: Wild-type
M-MLV: Moloney murine leukaemia virus
PAGE: Polyacrylamide gel electrophoresis
CCK-8: Cell counting Kit-8
PI: Propidium iodide
PS: Phosphatidylserine
NC: Negative control
AGO: Argonaute
FXR1: Fragile X mental retardation-related protein 1
AREs: AU-rich elements
