# Supplementary figures and images for: Upregulation of miR-205 induces CHN1 expression, which is associated with the aggressive behaviour of cervical cancer cells and correlated with lymph node metastasis
Source: BMC Cancer. 2020 Oct 27;20:1029. doi: 10.1186/s12885-020-07478-w (PMC7590479; doi:10.1186/s12885-020-07478-w)

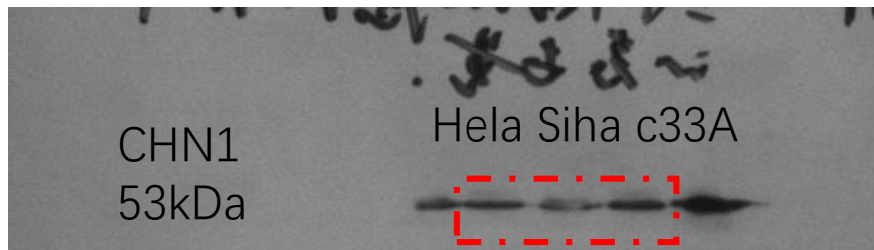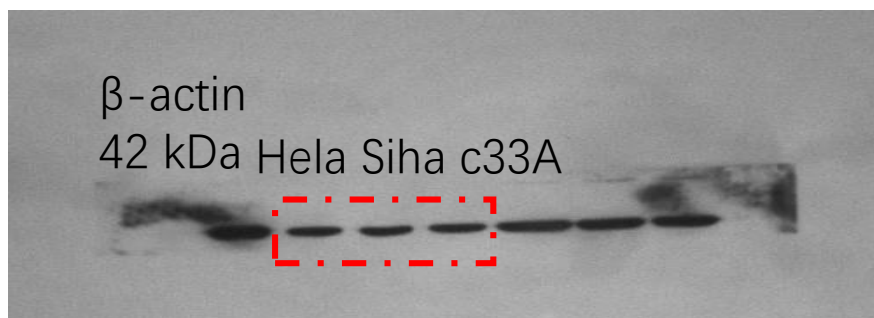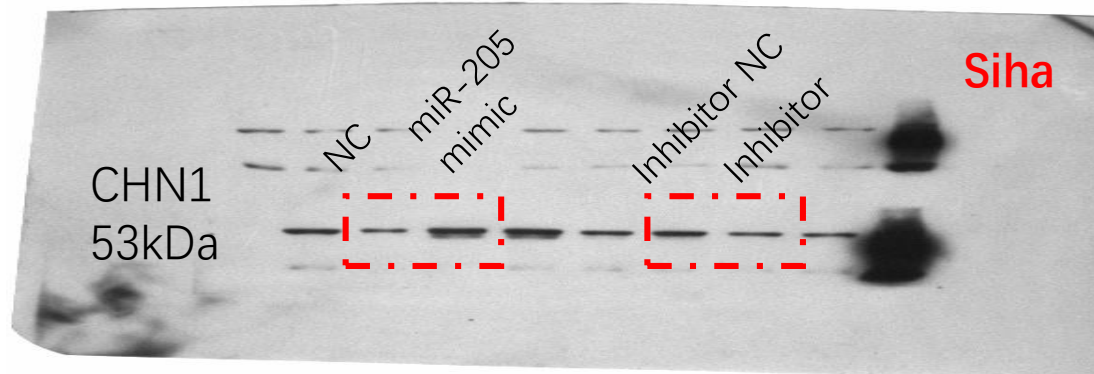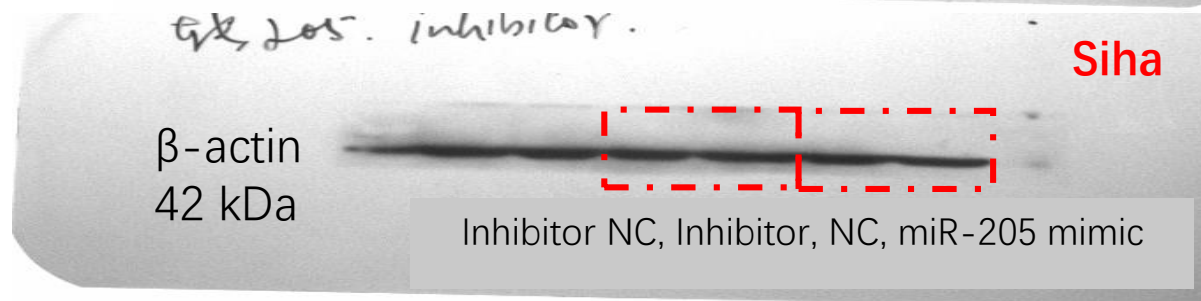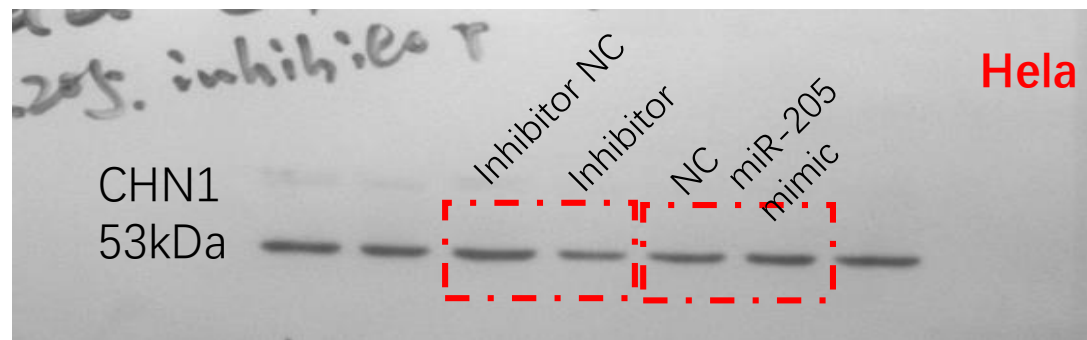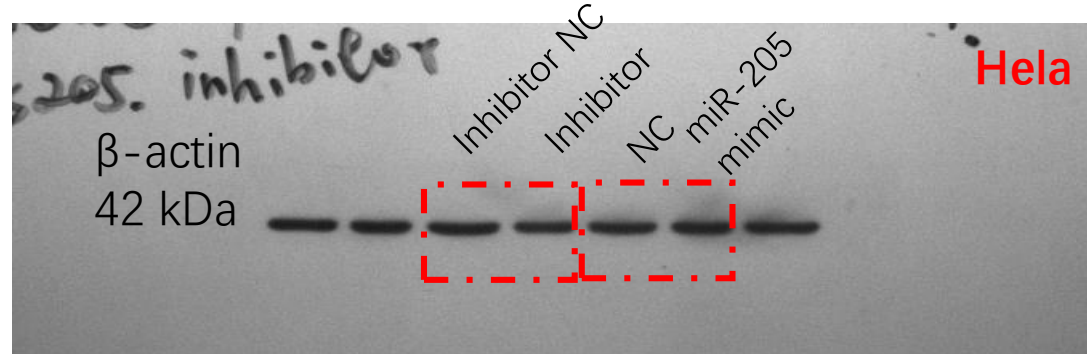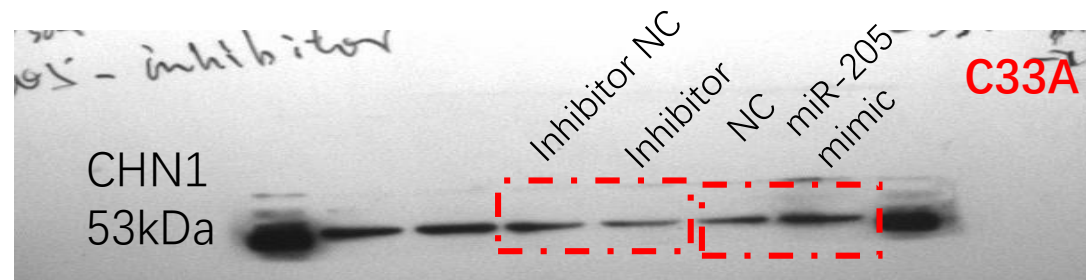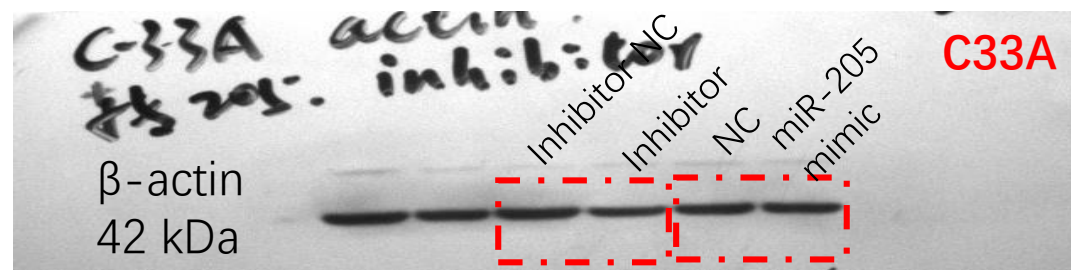

Supplement: Supplementary file 1 — Additional file 1 : Supplementary Figure 1. The original drawing of the cropped blots in the Fig. 3. Red dashed line showed where the cut was made. [file 12885_2020_7478_MOESM1_ESM.pdf]

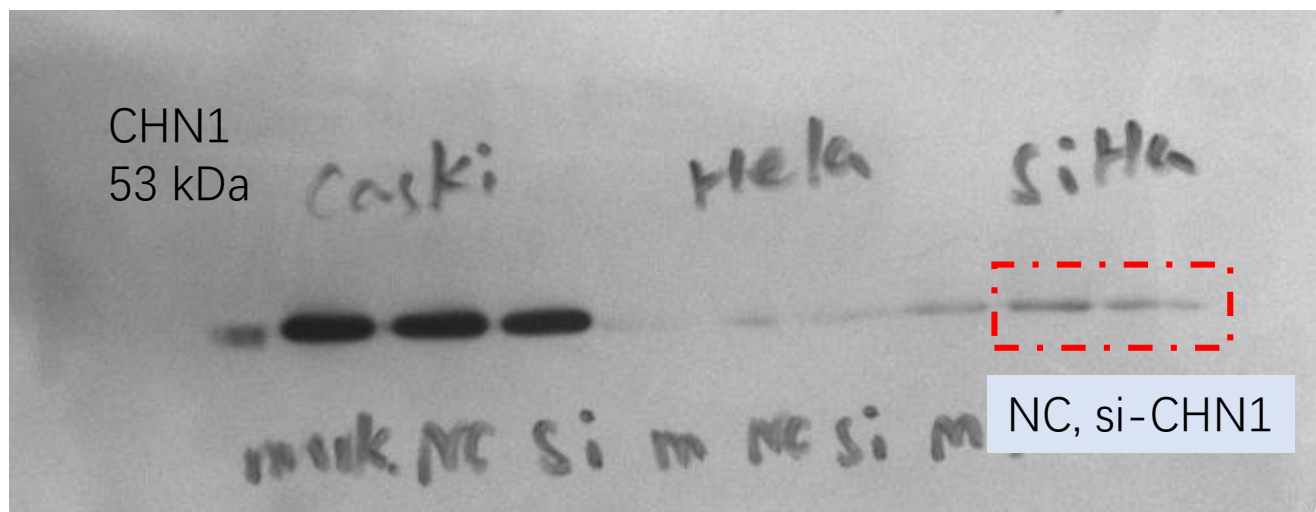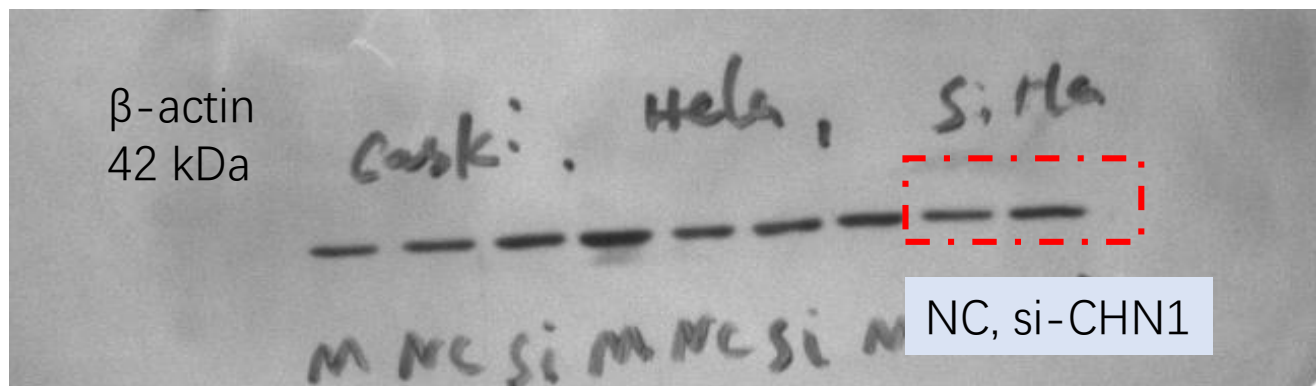

Supplement: Supplementary file 2 — Additional file 2 : Supplementary Figure 2. The original drawing of the cropped blots in the Fig. 7. Red dashed line showed where the cut was made. [file 12885_2020_7478_MOESM2_ESM.pdf]
